# Impact of Digital Media on the Patient Journey and Patient-Physician Relationship Among Dermatologists and Adult Patients With Skin Diseases: Qualitative Interview Study

**DOI:** 10.2196/44129

**Published:** 2023-09-22

**Authors:** Teresa Sofie Schick, Lea Höllerl, Tilo Biedermann, Alexander Zink, Stefanie Ziehfreund

**Affiliations:** 1 Department of Dermatology and Allergy School of Medicine Technical University of Munich Munich Germany; 2 Division of Dermatology and Venereology Department of Medicine Solna Karolinska Institute Stockholm Sweden

**Keywords:** digital media, dermatology, patient journey, patient-physician relationship, semistructured interview, qualitative content analysis

## Abstract

**Background:**

Digital media are easily accessible without time restrictions and are widely used for health- or disease-related purposes. However, their influence on the patient journey and the patient-physician relationship has not yet been sufficiently investigated.

**Objective:**

This qualitative interview study was designed to explore dermatologists’ and patients’ experiences with digital media for medical purposes in the context of patient journeys and patient-physician relationships.

**Methods:**

Twenty-eight semistructured video conference–based interviews were conducted and audiorecorded by experienced interviewers between November 2021 and June 2022 in Germany. Eligible patients were those who were aged ≥18 years, were affected by at least one physician-confirmed skin disease, and were fluent in the German language. The eligibility criterion for dermatologists was that they were currently practicing dermatology in an outpatient setting or in a hospital. Randomly selected dermatologists from the listing of the German National Association of Statutory Health Insurance Physicians and dermatologists from personal academic and professional networks were invited for participation via postal mail and asked to identify potential patient volunteers from their patient bases. All recorded data were pseudonymized, fully transcribed verbatim, and subsequently analyzed according to Mayring’s qualitative content analysis by 2 researchers, allowing for both a qualitative interview text analysis and a quantitative assessment of category assignments.

**Results:**

In total, 28 participants were interviewed: 16 adult patients and 12 dermatologists. Eight main categories emerged as key areas of interest: (1) the search for diagnosis and symptom triggers, (2) preconsultation digital media use, (3) in-depth information and exchange with other patients, (4) self-treatment, (5) patient-physician interaction, (6) roles of dermatologists and patients, (7) patient eHealth literacy, and (8) opportunities and risks. Categories 1 and 2 were only coded for patients; the other categories were coded for both patients and dermatologists. Patients reported searches for diagnosis or treatment options were most frequently (8/16) caused by a mismatch of symptoms and diagnosis or dissatisfaction with current therapies. Concerns regarding a potentially severe diagnosis prompted searches for initial or in-depth information before or after dermatological consultations. However, the large volume of information of varying quality often confused patients, leading dermatologists to assume the role of evaluating information from preinformed patients. Dermatologists generally encouraged the use of digital media, considered teledermatology advantageous, and viewed big data and artificial intelligence as being potentially beneficial, particularly when searching for rare diagnoses. A single, easily accessible, and free-of-charge platform with high quality information in lay language was recommended by the dermatologists and desired by patients.

**Conclusions:**

Digital media are widely accepted by both patients and dermatologists and can positively influence both the dermatological patient journey and patient-physician relationship. Digital media may therefore have great potential to improve specialized health care if patients and dermatologists embrace their new roles.

## Introduction

Digital media are easily accessible without time restrictions and are thus used by many people to seek information that will help them to understand their health conditions [1]. Throughout the last decades, digital media have grown in relevance for health purposes [2,3]. For example, the number of patients using the internet as a source of health information is steadily increasing [4]. Additionally, a variety of digital tools, such as telemedicine [5], remote monitoring [6], electronic medical records connected with patient portals [7,8], and health apps and wearables [9], have been designed to assist patients in managing their health care and in receiving the services they need [10].

The patient journey is patients’ experience of the various stages that they go through, from the first observation of disease signs or symptoms through medical consultation to final diagnosis and disease management. The reconstruction of a patient journey has been shown to be beneficial [11] since it may give insight into a patient’s perspective and experience [12,13], particularly the trigger events, initial health care contact, care, treatment, lifestyle changes, and ongoing care, revealing every facet of interaction, including the patient-physician relationship, between a patient and actors in the health care system [14]. This helps identify any gaps in patient care experience and provides an opportunity to redesign patient care to maximize clinical efficiency by targeting the activities most appreciated by patients [15]. While exemplary patient journey maps have recently been adapted for various noncommunicable diseases [13,14], no mapping has been presented that includes digital media use. Moreover, a recent review of 42 papers on internet-based health information seeking (with none from Germany) suggests that it can have a positive impact on the patient-physician relationship provided that eHealth literacy and the quality of online information are improved [16]. However, the influence of online health information seeking on patient-physician relationships appears quite complex [16] and still requires further investigation.

The medical specialty of dermatology appears to be particularly suitable for assessing the impact of digital media on the patient journey and the patient-physician relationship, considering the primarily visual manifestation of most dermatologic diseases [17], which allows patients with skin issues to compare their own skin findings with photographs and videos available online [18]. Additionally, skin diseases are the fourth most common cause of human illness [19]. Approximately one-third of the world’s population is affected by skin diseases [20], resulting in 57.4 million disability-adjusted life years in 2016 [21].

As no qualitative study has examined the effect of digital media on patient journeys and patient-physician relationships among outpatients and dermatologists, this study was designed to explore dermatologists’ and patients’ experiences with digital media use for medical purposes to (1) investigate the extent and purpose of digital media use among patients with skin diseases, (2) determine relevant aspects of digital media use regarding the patient journey and patient-physician interactions, and (3) identify opportunities and risks of digital media use from both a patient’s and dermatologist’s point of view.

## Methods

### Study Design

The established standard for reporting qualitative research, the COREQ (Consolidated Criteria for Reporting Qualitative Research) checklist, was followed in this study [22]. Semistructured interviews with dermatology patients and dermatologists from Germany were conducted via video conference–based meetings (Webex Meetings, Cisco Systems, Inc) between November 2021 and June 2022. Eligibility criteria for patients were (1) being aged 18 years or older, (2) being affected by at least one physician-confirmed skin disease, and (3) being fluent in the German language. The sole eligibility criterion for dermatologists was that they were currently practicing dermatology in an outpatient setting or in a hospital.

### Ethical Considerations

The study was reviewed and approved by the ethics committee of the medical faculty at Technical University of Munich (reference 266/21 S-EB) and conducted in accordance with the ethical standards of the Declaration of Helsinki. Participants were informed about the nature of the study, the pseudonymization of the data, and their rights as participants in lay terms; any questions regarding the study were answered before written informed consent documents were signed by both participants and interviewers. Data were anonymized during the transcription process.

### Recruitment and Interview Groups

A random sample of 100 dermatologists from the listing of the German National Association of Statutory Health Insurance Physicians (the Kassenärztliche Bundesvereinigung) were invited by postal mail to participate. The sample was generated without replacement by using a random generator sample in R (version 4.0.4; R Core Team), and a random seed was set for reproducibility as follows: set.seed(98765). Furthermore, personal academic and professional networks, including the Digital Dermatology Working Group of the German Dermatological Society (Deutsche Dermatologische Gesellschaft), were approached to recruit additional dermatologists.

Dermatologists were asked to identify potential patient volunteers from their patient bases. Snowball recruitment was used to include additional patients. In the patient sample, we recorded gender, age, disease duration, and skin disease, while for the dermatologists, we recorded gender and duration of professional experience. Video conference–based interview appointments were scheduled, and each participant received a pseudonym (D*x* for dermatologists and P*y* for patients, with *x* and *y* designating the numbers in chronological order of the interviews).

### Data Collection

Semistructured interview guides with open-ended questions were designed by the research team (TSS, SZ, and AZ) based on the relevant literature on patient journeys and patient-physician relationships and according to the manual for conducting qualitative interviews by Helfferich [23] (Multimedia Appendices 1 and 2). One interview guide was developed for patients with skin disease, focusing on three stages of the patient journey: (1) before, (2) during, and (3) after consultation. A second interview guide was prepared for dermatologists with a focus on patient-physician interactions. Each interview guide was pretested by a patient and a dermatologist and finalized according to their feedback.

Interviews were conducted in the German language by TSS and SZ (14 patients for TSS and 2 patients for SZ; 12 dermatologists for TSS), both of whom are female and have previous experience in qualitative research. The interviews were audiorecorded with the camera turned off unless otherwise requested by the interviewee. Interviews were conducted with no other person present. The interviewers did not know any of the participants prior to the study, and there were no repeat interviews. The interviewees were aware of the research aims and the name and qualifications of the researcher.

To obtain information on the distribution of interviewee characteristics, we recorded gender, age, and the skin disease of patient participants, whereas for dermatologists we only noted gender and workplace type (ie, medical practice, outpatient setting, or hospital). After each interview, field notes were made by the interviewers.

Data saturation in both groups was considered to be reached as soon as no additional new information appeared to be obtainable according to the subjective judgment of the interviewers [24,25]. Nevertheless, 2 more interviews were conducted in each group before interview-based data collection was terminated.

All 28 audio-recorded interviews were pseudonymized with the P*x* and D*x* pseudonyms for patients and dermatologists, respectively, with participant permission as declared by their written informed consent. The interviews were fully transcribed verbatim by a professional transcription (TranskriptionsSpezialist) service provider under strict privacy guidelines and checked by the interviewers. The transcripts were not returned to the participants.

### Data Analysis

Qualitative content analysis of the interviews was performed as previously described by Mayring [26], consisting of (1) development and application of deductive categories and (2) formation of inductive categories directly derived from the available interview content [27].

To obtain a category system that was sufficiently comprehensive and adequate for the purpose of the study [27], relevant topics were deductively derived from the interview guides (Multimedia Appendix 3, Tables S1 and S2) and were discussed and agreed upon between TSS, SZ, and AZ. Subsequently, 11 transcripts from the 28 interviews, including 5 dermatologists and 6 patients, were randomly selected by the interviewers (TSS and SZ), as suggested by Mayring [26], to inductively identify further relevant topics for the final category system by working through the transcripts line-by-line [26] and by independently modifying, specifying, and removing categories and discussing specific code attributions in depth.

The consolidated category system (Table 1) was then used by TSS and SZ as a basis to subsequently categorize the content of all interviews line by line with the qualitative data analysis software MAXQDA (2022 version, VERBI Software) for evaluation.

The remaining 17 of the 28 interviews were coded individually (7 dermatologists by SZ and 10 dermatology patients by TSS), and no further categories evolved during this process.

Based on the textual material in the transcripts, units of meaning formed units of analysis. Relevant content of the units of meaning was paraphrased to generate a category label. In accordance with the methodological literature [26], a low level of abstraction was initially selected and scaled down in the course of analysis and further review of the transcriptions. Subsequently, the abstraction level of the categories was harmonized to reach a uniform abstraction level of the category system. Text segments were recoded if necessary. Finally, quotes were selected to illustrate each category and its related subcategories and translated from German into English by a native speaker in both English and German.

**Table 1:** Main topics and associated categories for qualitative content analysis deductively derived from the interview guides and inductively categorized through line-by-line analysis of the interview material.

Topics	Categories
Use of digital media and purpose of use	C1: Search for diagnosis and symptom triggers C2: Preconsultation digital media use C3: In-depth information and exchange with other patients C4: Self-treatment
Digital media and patient-physician relationship	C5: Patient-physician interaction C6: Roles of dermatologists and patients
Digital media—influencer or assistant of the patient journey?	C7: Patient eHealth literacy C8: Benefits and risks

## Results

### Overview

A total of 28 interviews with an average duration of 14 (range 7 to 26) minutes were conducted, with 16 interviews conducted with dermatology patients (4 men, 12 women; median age 28, IQR 23.75-31.25 years) and 12 with dermatologists (8 men, 4 women; Table 2). Interview information about patient use of digital tools obtained from both dermatology patients and dermatologists is reported as the number of category assignments according to Tables 3-5.

Eight main categories emerged as key areas of interest: (1) search for diagnosis and symptom triggers, (2) preconsultation digital media use, (3) in-depth information and exchange with other patients, (4) self-treatment, (5) patient-physician interaction, (6) roles of dermatologists and patients, (7) patient eHealth literacy, and (8) benefits and risks. Categories 1 and 2 emerged only for patients, while the other categories emerged for both patients and dermatologists.

**Table 2:** Characteristics of participating patients and dermatologists.

Characteristics				Values
**Patients (n=16)**				
	Gender (female), n (%)			12 (75)
	Age (years) median (IQR)			28 (23.75-31.25)
	**Diagnosis, n (%)**			
		Acne	3 (19)	
		Atopic dermatitis	4 (25)	
		Acne and atopic dermatitis	1 (6)	
		Autosomal recessive congenital ichthyosis	1 (6)	
		Epidermolysis bullosa junctionalis	1 (6)	
		Psoriasis	1 (6)	
		Shingles	1 (6)	
		Skin irritations	2 (12)	
		Skin irritations and allergy	1 (6)	
		Urticaria	1 (6)	
**Dermatologists (n=12)**				
	Gender (female), n (%)			4 (33)
	**Setting, n (%)**			
		Hospital	10 (83)	
		Medical practice, outpatient setting	2 (17)	

**Table 3:** Quantitative analysis of qualitatively coded categories (categories 1-4) related to digital media use in 16 patients with skin diseases.

Categories	Codings, n
C1: Search for diagnosis and symptom triggers (including rationale for research of information and self-diagnosis)	26
C2: Preconsultation digital media use (including scheduling of appointments)	23
C3: In-depth information and exchange with other patients	20
C4: Self-treatment	38

**Table 4:** Quantitative analysis of qualitatively coded categories (categories 5 and 6) related to digital media use and the patient-physician relationship in 16 patients with skin disease and 12 dermatologists.

Categories	Patient codings, n	Dermatologist codings, n
C5: Patient-physician interaction (including online consultation, conversation, nonverbal communication, verbal communication, recommendations of digital tools by dermatologists, noncommunication)	34	64
C6: Roles of dermatologists and patients (including patient empowerment and participation)	16	57

**Table 5:** Quantitative analysis of qualitatively coded categories (categories 7 and 8), including subcategories related to digital media use, eHealth literacy, and opportunities and risks in 16 dermatology patients and 12 dermatologists.

Categories	Patients codings, n	Dermatologist codings, n
C7: Patient eHealth literacy	29	21
C8: Opportunities and risks (including adherence)	22	66

### Use of Digital Media by Patients With Skin Disease and Patient Journey

#### Search for Diagnosis and Symptom Triggers

To identify the most likely diagnosis, 7 of 16 patients with skin diseases reported using the internet to search for visible skin signs and disease symptoms using either keywords or pictures.

Patients entered into the Google search engine search phrases like “pimples in the face” (P1) or “reddish, stabbing spots” (P4) or searched for “triggers” (P2). Another patient reported his “fear that it might be something severe” (P13) as a reason for his web search.

Two patients who had been already diagnosed by dermatologists felt that the established diagnoses did not match their most recent or painful symptoms. Three patients reportedly used Google as a source of information while waiting for a dermatological appointment.

#### Preconsultation Digital Media Use

From the dermatologists’ point of view, patients using digital media before consultation were often highly concerned about their symptoms, as they suspected severe or frightening diagnoses that they had read about or seen pictures of. These sometimes “overinformed” (D12) patients consequently felt a strong urge to see a physician.

Digital images of a patient’s skin were also presented to the dermatologists during consultations via smartphone or by email, occasionally to emphasize an urgent need for consultation or to show the peak of the disease. One of the dermatologists asked for photographs prior to the appointment for preliminary evaluation, whereas another dermatologist expressed his disappointment that some patients did not bring any images to their appointment.

Patients reported that the possibility of using the internet to find nearby dermatologists, evaluate physicians, and schedule appointments (eg, via the Doctolib platform) was of high relevance to them: “I think online scheduling of an appointment is awesome, because you can easily...search for a dermatologist who is available next...” (P16).

#### In-Depth Information and Exchange With Other Patients

Collecting in-depth information following specialist consultations was also considered useful from the point of view of the patients and one of the dermatologists, as some information might be missed by the patient during the relatively short consultation time. Patients looked up information on, for example, their diagnosis or skin disease in general, medical terminology, mode of action of the prescribed medication, personal experience of others receiving the same therapy, alternative therapies, and methods to promote the healing process.

Exchange with other patients who are similarly affected appears also to be very important from both patient and dermatologist perspectives, “because...patients in general have high competencies living with their disease in everyday life” (P6). Both physicians and patients emphasized this, especially when the skin disease was rare.

#### Self-Treatment

Patients most frequently reported the use of digital media in searching for self-treatment options or alternatives because of dissatisfaction with their dermatological treatment, side effects from their prescribed medical treatments, long waits for dermatological consultation, and psychological strain or stress due to their disease. The majority mentioned web search engines and social media platforms as relevant sources for information about self-treatment options. Patients reported multiple trials of self-treatment, such as with various creams or ointments and homeopathic medicine. Although a few patients also reported some positive improvements in their skin condition, most of the patients reported failure of their self-treatment attempts.

### Digital Media and Patient-Physician Interaction

#### Patient-Physician Interaction

Depending on the availability of time and the complexity or severity of their patient’s skin disease, most dermatologists reported that they inquired about their patient’s internet use to better understand their current “knowledge and expectation” (D4). However, some dermatologists would not directly ask their patients to prevent them from “feeling ashamed” (D5) or even “apologizing for it” (D5).

Generally, the dermatologists recommended to their patients digital information channels that were predominantly self-help groups and less frequently websites containing information about medical guidelines, especially to patients with certain skin conditions and with adequate eHealth literacy. For patients with chronic diseases, it was recommended “to google everything and take notes of their questions” (D1), which could then be discussed during consultation. Some dermatologists, however, reported that they would not recommend digital tools or websites at all:

“Patients come to visit us, to receive information and practical guidance regarding their disease. I think it is the main part of my profession to advise them and not to refer them elsewhere.” (D6)

Except for one patient, none of the patients received any recommendations for digital information sources from their dermatologists, even though recommendations from a dermatologist would have been considered more trustworthy.

While 2 patients affected by acne after unsuccessful treatments (self-treatments) had paid for asynchronous dermatological online consultations with a German dermatological telemedicine company, none of the interviewed patients had attended a synchronous teledermatological consultation. A few dermatologists reported that they offered asynchronous or synchronous teledermatology, or both, and appreciated the flexibility of working hours allowed for by asynchronous telemedicine and the potential to increase efficiency in patient care and avoid frustration on both sides: “The problem is that while patients are waiting for an appointment, the skin may change through therapies or non-treatment, so that it’s sometimes really difficult to judge” (D4). One dermatologist felt that telemedicine uses up time no longer available for “live patients” (D8).

#### Roles of Dermatologists and Patients

Almost all dermatologists reported that they considered it part of their profession to “classify” (D9) and “evaluate” (D5) online health information that patients address during consultations. This included “resolving or clarifying patients’ mistaken self-diagnoses or presumptions” (D3), often leading to longer communication.

One dermatologist explained that “a certain openness on both sides” (D4) is needed during consultation, that is, the physician needs to be responsive to the patient’s information and the preinformed patient needs to trust the physician’s competence. Accordingly, the majority of the dermatologists appreciated preinformed patients, and one dermatologist highlighted that “it will save time...and ease agreements on therapy if patients are well informed” (D9). Dermatologists therefore expressed a need to improve their own skills regarding digital tools, particularly in the fields of informatics and artificial intelligence (AI) or digital health applications.

### Digital Media—Influencer or Assistant of the Patient Journey?

#### Patient eHealth Literacy

From the dermatologists’ perspective, they considered that patients varied strongly in their eHealth literacy, ranging from reflecting on digital media readings to “getting hysterical” (D6). While one dermatologist reported that patients often questioned what they had read on digital media and would rather “trust the physician” (D6), most dermatologists were concerned that patients were unable to identify misinformation and did “not have the health literacy to process all the information” (D12). Therefore, they suggested recommending suitable websites to their patients.

A number of patients considered the “huge amount” (P2) of digital health information as problematic regarding the trustworthiness of the sources. One patient reported having felt worried during his web search after having found diagnoses “ranging from less to very severe” (P13), while 3 patients with an academic background claimed that they had the media literacy to filter relevant and trustworthy information. Overall, a recent improvement in the quality of digital information has been reported by both dermatologists and patients.

#### Opportunities and Risks

From a dermatologist’s perspective, the market of health-related digital tools and information channels is “extremely confusing” (D12), and both patients and physicians “are flooded” (D12) with offers. Therefore, some expressed their desire for a single digital platform for various skin diseases providing understandable, high-quality content that is “neutral” (D11), “easily accessible...for free [and] developed from both physicians and patients to ensure that patient needs are met” (D1), and that they could recommend to their patients.

Clinical decision support systems and the use of AI or big data analytics were considered leading technologies by most dermatologists that would potentially allow them “to diagnose a rare disease at an earlier point” (D2). Half of the dermatologists considered the prospective use of health monitoring apps as beneficial for both themselves and their patients. Such apps could provide dermatologists and patients with information about the course of their disease or side effects and could include a diary feature to “promote [patients’] adherence to therapy” (D4). However, one dermatologist commented that “such apps bear the risk of incorrect measurements or misinterpretations...” (D2).

Most patients considered that teledermatological consultations represented significant progress with great potential in certain situations, such as when “quickly receiving the prescription and to immediately start therapy” (P14) or when the diagnosis is already known, thereby avoiding time-consuming “rides” (P9) to the doctor’s office. Other patients expressed their desire for a “best practice guide developed in Germany as is available in the United Kingdom” (P6) or a “scientific-based questionnaire” (P10) for establishing a self-diagnosis based on signs and symptoms.

## Discussion

This study provides in-depth insight into patient and dermatologist experiences with digital information-seeking behavior for medical needs and its impact on health care, thus presenting an evidence base for high-quality care in an increasingly digitized world. The interview study revealed that digital media are predominantly used by dermatology patients in their search for diagnoses, symptom triggers, and new or alternative treatment options because of dissatisfaction with current therapies or a desire for therapeutic options with fewer side effects. Furthermore, a personal motivation of the patients to be well-informed about their underlying disease was observed. Moreover, the availability of teledermatology was valued as a novel technical facilitator by both patients and dermatologists. A potentially positive influence on the patient-physician relationship could also be identified from both patients’ and the dermatologists’ perspectives, as indicated by a dermatologist’s suggestion “to google anything and take notes” (D1), which may then be discussed during consultation. From a patient’s perspective, recommendations for digital media by their dermatologist were considered most trustworthy.

The results are in line with recently published findings from a quantitative study on dermatology patients in Switzerland that reported that self-diagnosis, self-therapy, search for alternative therapies, better understanding of the disease, and interactions with other patients were the primary motivators for patient digital media use [3]. Another quantitative study on dermatology patients in the United States showed that another motivation was to potentially avoid the necessity for professional consultation [28]. The qualitative approach of our study made it possible to unveil additional important facets of the patient journey that many patients experience during their long waits for medical appointments.

The large amount of health information available from digital media, ranging from personal opinions in forums to medical literature, was considered challenging and sometimes even confusing for patients in the study, which is in accord with previous reports [29-31]. While AI and big data analysis have already proven to be suitable tools for finding correct diagnoses in certain situations [32], some of the information digitally retrieved from the internet may be qualitatively less useful. Therefore, the relevance of patient eHealth literacy is further emphasized by these findings; this has been previously stressed as an important issue [4,29] to prevent patient misinformation, confusion, or distress [29]. In this context, both dermatologists and patients recommended the use of a single, evidence-based, high-quality information platform for skin diseases that is easily accessible at no cost for patients. Such a platform [33] was reported to reduce the demand for primary care for minor conditions by providing information on appropriate self-care [34]. This points to the potential of chatbots as a novel and easily available application of medical expertise for patients and physicians and suggests that they should definitely be considered as an integral part of such a platform.

In a previously published interview study, family physicians reported that they experienced consultations with patients who had informed themselves on the internet as demanding because of the additional responsibility to contextualize and interpret their patients’ web-sourced health information [29]. While the results of this study also indicate that the classification and evaluation of web-based information can increase consultation time, this additional task was perceived as an integral part of a dermatologist’s professional role. In our study, dermatologists valued preinformed patients for their higher level of knowledge during discussions of treatment options.

This study has several strengths and limitations. Because of the COVID-19 pandemic, the interviews were carried out via video calls, which could have had an effect on the reliability of the data. However, in-person interviews are assumed to be only slightly superior to video calls [35]. In addition, a possible selection bias should be considered; this could have arisen from the fact that participants were more interested in digitalization in medicine than nonparticipants. This could also explain why teledermatological consultations appeared to be widely available on the dermatologists’ side, despite being not widely offered to participating patients. However, to understand the impact of digital media on the patient journey and the patient-physician relationship, it was useful to interview participants who were familiar with digitalization in medicine. Prior studies have found that poor local language competency and being a member of an ethnic minority are negatively associated with accessing internet-based health information [36,37]; thus, the inclusion of only patients fluent in the German language has to be considered. Nevertheless, the patients had highly heterogeneous skin diseases and disease duration, and although gender and age heterogeneity were not fully achieved, the views of women and men, as well as younger and older patients and dermatologists, were included. Given the qualitative character of the study, the findings cannot be generalized. Having said that, an adequate sample size and content saturation were reached in this study [25]. Moreover, investigations of digital media or internet use for health-related information predominantly use surveys for quantitative data analysis [4,16]. Since use of a predefined set of questions may overlook subjective opinions or experiences of study participants, semistructured interviews were chosen for this study. Qualitative content analysis was subsequently applied to integrate both qualitative and quantitative steps of analysis [27], thereby providing supportive analytic power to the results of this study.

To further investigate the influence of digital media use on the 3 stages of the patient journey (before, during, after consultation) as well as on the patient-physician relationship, a quantitative study should be performed with a larger sample of patients with skin disease. Since most of the participating dermatologists in this study were practicing in a hospital setting, a future quantitative study should include dermatologists employed in outpatient settings.

Future research should also be directed toward the development of a patient-centered digital health information platform for skin diseases, with a particular focus on patient needs and expectations. This may assist in addressing the issue of improving patient eHealth literacy.

Our study demonstrates that digital media are accepted by both patients and dermatologists and are able to positively influence all stages of a dermatological patient journey, as well as the patient-physician relationship. Thus, digital media have great potential to improve specialized health care. However, efforts must be undertaken to ensure comprehensive eHealth literacy in the general population and to increase the availability of evidence-based and trustworthy resources in digital media.

## Appendix

Multimedia Appendix 1 Original interview guide for patients translated into English.

Multimedia Appendix 2 Original interview guide for dermatologists translated into English.

Multimedia Appendix 3 Supplementary Tables S1 (Items of interest derived from the interview guide for patients with skin diseases addressing digital media use in relation to the stage of the dermatological patient journey including opportunities and risks) and S2 (Items of interest derived from the interview guide for dermatologists addressing patient digital media regarding patient-physician interactions).

## Abbreviations

AI: artificial intelligence
COREQ: Consolidated Criteria for Reporting Qualitative Research
Dx: dermatologist
Py: patient

## Data Availability

The data sets generated during and/or analyzed during this study are available from the corresponding author on reasonable request.

## References

[1] Bach RL, Wenz A (2020). Studying health-related internet and mobile device use using web logs and smartphone records. PLoS One.

[2] Mehta-Ambalal SR, Nisarta M (2021). Dermatology 2.0- How the internet is changing us, our patients and our practice. Indian Dermatol Online J.

[3] Gantenbein L, Navarini AA, Maul LV, Brandt O, Mueller SM (2020). Internet and social media use in dermatology patients: Search behavior and impact on patient-physician relationship. Dermatol Ther.

[4] Tan SS, Goonawardene N (2017). Internet health information seeking and the patient-physician relationship: A systematic review. J Med Internet Res.

[5] Glines KR, Haidari W, Ramani L, Akkurt ZM, Feldman SR (2020). Digital future of dermatology. Dermatol Online J.

[6] Peyroteo M, Ferreira IA, Elvas LB, Ferreira JC, Lapão Luís Velez (2021). Remote monitoring systems for patients with chronic diseases in primary health care: Systematic review. JMIR Mhealth Uhealth.

[7] Dendere R, Slade C, Burton-Jones A, Sullivan C, Staib A, Janda M (2019). Patient portals facilitating engagement with inpatient electronic medical records: A systematic review. J Med Internet Res.

[8] Kim J, Ryu B, Cho S, Heo E, Kim Y, Lee J, Jung SY, Yoo S (2019). Impact of personal health records and wearables on health outcomes and patient response: Three-arm randomized controlled trial. JMIR Mhealth Uhealth.

[9] Paradis S, Roussel J, Bosson J, Kern J (2022). Use of smartphone health apps among patients aged 18 to 69 years in primary care: Population-based cross-sectional survey. JMIR Form Res.

[10] Affinito L, Fontanella A, Montano N, Brucato A (2020). How physicians can empower patients with digital tools. J Public Health (Berl.).

[11] Trebble TM, Hansi N, Hydes T, Smith MA, Baker M (2010). Process mapping the patient journey: an introduction. BMJ.

[12] Wolterbeek N, Hiemstra DJ, van der Hoeven FA, Auw Yang KG (2019). Using patient experience in optimizing the total knee arthroplasty patient journey. Patient Exp J.

[13] Kuo S, Huang KE, Davis SA, Feldman SR (2015). The rosacea patient journey: a novel approach to conceptualizing patient experiences. Cutis.

[14] Devi R, Kanitkar K, Narendhar R, Sehmi K, Subramaniam K (2020). A narrative review of the patient journey through the lens of non-communicable diseases in low- and middle-income countries. Adv Ther.

[15] McCarthy S, O’Raghallaigh P, Woodworth S, Lim YL, Kenny LC, Adam F (2016). An integrated patient journey mapping tool for embedding quality in healthcare service reform. J Decis Syst.

[16] Luo A, Qin L, Yuan Y, Yang Z, Liu F, Huang P, Xie W (2022). The effect of online health information seeking on physician-patient relationships: Systematic review. J Med Internet Res.

[17] George DD, Wainwright BD (2012). Dermatology resources on the internet. Semin Cutan Med Surg.

[18] AlGhamdi KM, Almohideb MA (2011). Internet use by dermatology outpatients to search for health information. Int J Dermatol.

[19] Hay RJ, Johns NE, Williams HC, Bolliger IW, Dellavalle RP, Margolis DJ, Marks R, Naldi L, Weinstock MA, Wulf SK, Michaud C, J L Murray C, Naghavi M (2014). The global burden of skin disease in 2010: an analysis of the prevalence and impact of skin conditions. J Invest Dermatol.

[20] Bickers DR, Lim HW, Margolis D, Weinstock MA, Goodman C, Faulkner E, Gould C, Gemmen E, Dall T, American Academy of Dermatology Association, Society for Investigative Dermatology (2006). The burden of skin diseases: 2004 a joint project of the American Academy of Dermatology Association and the Society for Investigative Dermatology. J Am Acad Dermatol.

[21] GBD 2016 DALYs and HALE Collaborators (2017). Global, regional, and national disability-adjusted life-years (DALYs) for 333 diseases and injuries and healthy life expectancy (HALE) for 195 countries and territories, 1990-2016: a systematic analysis for the Global Burden of Disease Study 2016. Lancet.

[22] Tong A, Sainsbury P, Craig J (2007). Consolidated criteria for reporting qualitative research (COREQ): a 32-item checklist for interviews and focus groups. Int J Qual Health Care.

[23] Helfferich C (2011). Die Qualität qualitativer Daten: Manual für die Durchführung qualitativer Interviews.

[24] Guest G, Bunce A, Johnson L (2016). How many interviews are enough?. Field Methods.

[25] Saunders B, Sim J, Kingstone T, Baker S, Waterfield J, Bartlam B, Burroughs H, Jinks C (2018). Saturation in qualitative research: exploring its conceptualization and operationalization. Qual Quant.

[26] Mayring P, Brunner E, Buber R, Holzmüller HH (2008). Qualitative inhaltsanalyse. Qualitative Marktforschung.

[27] Mayring P, Bikner-Ahsbahs A, Knipping C, Presmeg N (2015). Qualitative content analysis: Theoretical background and procedures. Approaches to Qualitative Research in Mathematics Education.

[28] Wolf JA, Moreau JF, Patton TJ, Winger DG, Ferris LK (2015). Prevalence and impact of health-related internet and smartphone use among dermatology patients. Cutis.

[29] Ahmad F, Hudak PL, Bercovitz K, Hollenberg E, Levinson W (2006). Are physicians ready for patients with Internet-based health information?. J Med Internet Res.

[30] Ahluwalia S, Murray E, Stevenson F, Kerr C, Burns J (2010). 'A heartbeat moment': qualitative study of GP views of patients bringing health information from the internet to a consultation. Br J Gen Pract.

[31] Berland GK, Elliott MN, Morales LS, Algazy JI, Kravitz RL, Broder MS, Kanouse DE, Muñoz J A, Puyol JA, Lara M, Watkins KE, Yang H, McGlynn EA (2001). Health information on the Internet: accessibility, quality, and readability in English and Spanish. JAMA.

[32] Du-Harpur X, Watt FM, Luscombe NM, Lynch MD (2020). What is AI? Applications of artificial intelligence to dermatology. Br J Dermatol.

[33] Gann B (2012). Giving patients choice and control: health informatics on the patient journey. Yearb Med Inform.

[34] Murray J, Majeed A, Khan MS, Lee JT, Nelson P (2011). Use of the NHS Choices website for primary care consultations: results from online and general practice surveys. JRSM Short Rep.

[35] Krouwel M, Jolly K, Greenfield S (2019). Comparing Skype (video calling) and in-person qualitative interview modes in a study of people with irritable bowel syndrome - an exploratory comparative analysis. BMC Med Res Methodol.

[36] Samkange-Zeeb F, Borisova L, Padilla B, Bradby H, Phillimore J, Zeeb H, Brand T (2020). Superdiversity, migration and use of internet-based health information - results of a cross-sectional survey conducted in 4 European countries. BMC Public Health.

[37] Yoon H, Jang Y, Vaughan PW, Garcia M (2020). Older adults' internet use for health information: Digital divide by race/ethnicity and socioeconomic status. J Appl Gerontol.

