# Supplementary material for: Impact of Digital Media on the Patient Journey and Patient-Physician Relationship Among Dermatologists and Adult Patients With Skin Diseases: Qualitative Interview Study
Source: J Med Internet Res. 2023 Sep 22;25:e44129. doi: 10.2196/44129 (PMC10559188; doi:10.2196/44129)
Supplement: Multimedia Appendix 1 [file jmir_v25i1e44129_app1.docx]

**Supplementary material 1**

**Interview guide for patients**

| Welcoming | ... |
| --- | --- |
| Introduction of interviewer | ... |
| Clarification about the study & data protection | ... |
| Checking the inclusion & exclusion criteria | ... |
| Informed consent request | ... |

| **Demographic characteristics of the patient** |
| --- |
| Sex: female □ male □ other □  Age: ___________ years  Highest school degree/education^[[1]](#footnote-1)^: ________________________  Marital status: single □ in partnership □  Medical experience: yes □ no □ |
| **Health information of the patient** |
| Diagnosis:  Time of diagnosis:  Others: |

| **Definition of (Digital) Information Channels** |
| --- |
| An information channel, as defined by communications research, is a "transmission channel, medium through which information is disseminated, received, and exchanged."^^[[2]](#footnote-2)^^ Information channels create a broad base of information from print to radio to social media.^^[[3]](#footnote-3)^^ Offline channels^^[[4]](#footnote-4)^^  are for example posters, brochures or a personal conversation with acquaintances, doctor/pharmacist. Digital information channels extend the offline information offer via the electronic transmission path (radio or cable), such as digital health applications (apps) or online information portals^^[[5]](#footnote-5)^^. This also includes informal digital information channels, such as WhatsApp groups or similar digital communities^^[[6]](#footnote-6)^^. |

| **Open narrative of the patient at the beginning of the interview** |
| --- |
| To begin with, we would like you to tell me about your personal experience as a patient through your medical journey, i.e. from the onset of your first symptoms until today. We are particularly interested in which (digital) information channels you used at which point in time, which digital applications you used, and how this influenced your journey.  We will then go into some aspects in more detail. |

|  | **Main Question** | **Detailed Question** | **Aim** |
| --- | --- | --- | --- |
| I | Before medical consulation | | |
|  | **If presentation to dermatologist due to a skin change:**  **How did you become aware of your skin change?** |  | The aim is to find out the influencing factors that lead to the beginning of the Patient Journey:  e.g. the patient has recognized the skin change himself/herself, recurrence, read about it, talked to other patients, screening....  Ideally, the patient describes his/her journey from the first or repeated recognition of a skin change to the dermatologist and beyond. |
|  | **You have just described to me how you became aware of your skin change. Now I would be interested to know how you subsequently informed yourself about your health situation?** And that was before your first visit to the doctor. | Were there any points of contact, such as the pharmacy or your circle of acquaintances?  Which information channels did you use to find out about your complaints?  And which digital information channels or applications did you use to inform yourself about your complaints? | Identification of the information channels used by patients when searching for health information.  Information channels includes both.  (1) Internet-based applications, e.g., weblogs, podcast, symptom checker apps, as well as  (2) offline offerings, such as magazines, doctor, pharmacist. |
|  |  | What health information was most important to you at this stage? | Identification of health information that is interesting for patients and about which they want to learn more.  In addition, the subjective evaluation of the found health information will be asked. |
|  |  | How much time did you spend searching for health information at this stage? | Identification of a temporal pattern of use e.g. daily information search on blogs, one-time information search, etc. |
|  | **Why did you seek health information?** |  | Capture patients' motives for seeking information. The literature shows e.g.   - Better understanding of the disease - Self-diagnosis/therapy - Searching for alternative therapies - Anxiety - Interaction with other internet users - Distrust in physicians - To share experiences   Other e.g., online research by medical students, complementary information in addition to physician's advice) (L.Gantenbein 2020) |
|  | **How did you feel after your search for information?** | Which (digital) information channels or what evoked "those feelings" in you?  How satisfied are you with the health information you found?  What concerns do you have about searching for health information in digital information channels? | The aim is to find out how the patient feels after searching for information in digital information channels (e.g., reassured, worried, etc.).  At the same time, the aim is to find out how the patient assesses the information found. |
|  | **How did you come to the decision to consult a doctor?** | When was the time that you decided to consult a doctor?  What research in advance influenced this decision? | Identification of the point in time and the reasons in the Patient Journey at which the patient decides to see a (skin) doctor. |
| II | *During consulation* | | |
|  | **You then decided to see a (skin) doctor, how did you proceed?** | Why did you choose this doctor?  How did you make your appointment?  Did you visit the practice or did you have a telemedical treatment (video consultation)?  In case of telemedical treatment: How did you experience this treatment? | Identification of factors that influence the choice of the doctor and the framework of the medical appointment. |
|  | **What health information have you found that you have talked about with your doctor?** | What health information have you discussed together with your dermatologist(s)? | Identify the health information from digital information channels that comes up in a doctor-patient conversation and how it is addressed in the conversation. |
|  |  | *How has your dermatologist/doctor handled health information from your digital information channels/apps?* | The dermatologist's response to information from (digital) information channels/applications should be recorded. |
|  |  | *Which other digital information channels/applications did your dermatologist recommend to you during the interview?* | Identify digital information channels that dermatologists believe are suitable for information seeking. |
|  | **How did your prior information affect communication with your dermatologist(s)?** | What medical questions were you able to ask your dermatologist(s) based on your preliminary information?  Which medical statements of your dermatologist(s) were well understandable for you based on your prior information?  In which situation could you talk about the health information from (digital) information channels with your doctor(s)? | The aim is to understand the patient's point of view, to what extent his/her prior information has influenced the communication with the dermatologist and what positive or negative effects he/she sees in it.  The aim is to find out whether there were so-called "facilitators" during the doctor-patient conversation that facilitated communication about found health information from (digital) information channels.  In addition, it should be recorded how the patient felt in this situation.  In the literature e.g.  Patients discussed online findings during doctor visits when a family member was present.(Silver 2015) |
|  | *If prior information was not discussed in digital information channels:*  **What prevented you from talking about the information you found with your dermatologist?** | How did you feel during the interview with your dermatologist? | Identification of communication barriers: For example, the literature shows   - feared being perceived as challenging or confronting their physician. (Hay, Cadigan et al. 2008) - Patients experienced resistance from doctors over bringing information (Stevenson, Kerr et al. 2007) - Patients might feel being disapproved of by the physician if they shared their Internet search. (Broom 2005) - lack of time during consultations, or reluctance to interfere with the consultation process. (Sommerhalder, Abraham et al. 2009) - fear of embarrassment; feeling it would be insulting to the physician (Silver 2015) |
| III | After medical consultation | | |
|  | **How was your subjective perception after the face-to-face meeting with your doctor(s)?** |  | Understanding subjective perceptions of the patient with regard to the doctor-patient relationship  Trust  Comment:  E.g. patient is confused due to different information (doctor vs. prior information), patient is reassured, etc. |
|  | **How would you describe the trusting relationship you have with your physician?**  **Thinking back to your search for health information:**  **What health information from digital channels did you trust?** | Can you name the reasons why you trust your doctor(s)?  Depending on the patient's answer:  Why don't you trust your doctor?  Can you name the reasons why you trust this health information?  Depending on patient response:  Why do you not trust this health information? | "Googling is good - trust is better". The aim is to find out how the patient describes the relationship of trust with his or her doctor and what trust he or she places in digital information channels. |
|  | **What other health information did you look for after your doctor's appointment?** | Which statements (or technical terms) of your/your dermatologist did you search for in (digital) information channels following your medical appointment?  Which information channels/applications did you use for further search or treatment?  Why did you search for this information from your dermatologist after the personal consultation? | The aim is to find out which health/medical information the patient continues to search for in which (digital) information channels after his or her consultation with the dermatologist.  In addition, the reasons for this behavior are to be found out.  e.g. checking the diagnosis or therapy, getting a second opinion, not understanding technical terms, finding alternative therapies, exchanging information with other patients, etc. |
| IV | Future | | |
|  | **Where will you look for health information again in the future?** | Before which doctor visits will you use this approach of searching for information in (digital) information channels/applications again? | Identification of a repetitive pattern of search behavior in patients.  Possible transfer of the search behavior to other medical specialties. |

| **Summary & Final Question** |
| --- |
| Thank you for sharing your experience with us! I would like to ask you one final question:  If you now look back on your medical journey as a patient, i.e. from your first symptom search, to cause research, to your dermatologist and the specific treatment intervention:  What would you have liked to see along your path through your phases?  And what would you have liked from digital information channels/applications? |

We have now reached the end of the interview. Thank you very much for your participation.

1. ISCED (Internationale Standardklassifikation des Bildungswesens) <https://www.datenportal.bmbf.de/portal/de/glossary-i.html> [↑](#footnote-ref-1)
2. <https://www.dwds.de/wb/Informationskanal>, Zugriff: 19.05.2021, 17:00 Uhr [↑](#footnote-ref-2)
3. <https://www.bundesgesundheitsministerium.de/fileadmin/Dateien/5_Publikationen/Ministerium/Broschueren/Im_Dialog_-_Ausgabe_5_.pdf>, Zugriff: 19.05.2021, 17:00 Uhr [↑](#footnote-ref-3)
4. <https://www.hss.de/download/publications/AA_71_Mittelpunkt_Buerger_02_neu.pdf>, Zugriff: 19.05.2021, 17:00 Uhr [↑](#footnote-ref-4)
5. <https://www.svr-gesundheit.de/fileadmin/Gutachten/Gutachten_2021/SVR_Gutachten_2021_online.pdf>, Zugriff:19.05.2021, 17:00 Uhr [↑](#footnote-ref-5)
6. <https://link.springer.com/content/pdf/10.1007/s00103-019-03078-7.pdf>, Zugriff:19.05.2021, 17:00 Uhr [↑](#footnote-ref-6)
