# Supplementary material for: Impact of Digital Media on the Patient Journey and Patient-Physician Relationship Among Dermatologists and Adult Patients With Skin Diseases: Qualitative Interview Study
Source: J Med Internet Res. 2023 Sep 22;25:e44129. doi: 10.2196/44129 (PMC10559188; doi:10.2196/44129)
Supplement: Multimedia Appendix 2 [file jmir_v25i1e44129_app2.docx]

**Supplementary material 2**

**Interview guide for dermatologists**

| Welcoming | ... |
| --- | --- |
| Introduction of interviewer | ... |
| Clarification about the study & data protection | ... |
| Checking the inclusion & exclusion criteria | ... |
| Informed consent request | ... |

| **Demographic characteristics of the dermatologists** |
| --- |
| Sex: female □ male □ other □  Age: ___________ years  Professional experience: ___________ years |

| **Thematic introduction** |
| --- |
| Nowadays, health information in digital information channels is basically available to anyone at any time of day with just one click. This rapidly increasing number of digital information in recent years also exerts an influence on the individual health path of a patient through all phases of his or her illness, i.e., from the initial search for symptoms to research into causes to concrete treatment measures (patient journey). In a publication of the New England Journal of Medicine, it is described that digital information offers pose great challenges to the relationship between doctor and patient, as patients use alternative (digital) information channels. |

| **Open narrative of the dermatologists at the beginning of the interview** |
| --- |
| What has changed in your daily practice due to digitally available health information and thus pre-informed patients? |

|  | **Main Question** | **Detailed Question** | **Aim** |
| --- | --- | --- | --- |
| I | *The physician's role perception* | | |
|  | **How would you describe your role as a physician?** | To what extent do you find that your role has changed because of the advancing digitization in in medicine?  To what extent do you find that your role changed by "pre-informed patients"? | Capture the theme of the recognizes changing of the role. Classic role understanding from sociology: "the good doctor". Is there a changing understanding of the role due to pre-informed patients? e.g., physician as translator of information or from physician to health coach. |
| II | *Dealing with pre-informed patients* | | |
|  | **How do you deal with pre-informed patients in your daily practice?** | Do you ask the patient if they have already sought information about their complaints?  If yes: To what extent do you also address the patient's information?  How do you experience your patients when you talk to them about their preliminary information?  If no: why don't you ask the patient? | Identification of the inclusion and handling of pre-informed patients.  Identification of the patient's emotional state, such as worried, self-confident, ready for discussion etc. |
|  | **What is your impression: Why do patients search for health information before they consulate a physician?** |  | Identification of search motives of patients from a physician's perspective. |
|  | **Can you describe in more detail the information-seeking patients who have already obtained information from digital channels before visiting the doctor?** | What influence do socioeconomic characteristics on the characteristics on search behavior? | The aim is to identify possible characteristics of the of the pre-informed patients such as possible age-, or gender-dependent factors, etc.  Is there one or more types of information-seeking patients? |
| III | *Assessment of the digital health literacy of patients* | | |
|  | *Digital health literacy (e-health literacy) describes the ability to find, understand, assess, and apply health-related information in relation to digital applications and information offerings.*  **Against this background, how do you against this background the digital health literacy of your patients?** |  | The aim is to gain an impression from a physician's perspective,  how the digital health literacy of patients is estimated. e.g., Do patients have difficulties finding the right information or understanding it? |
|  | **From your perspective, how does the pre-informed patient influence the patient-physician relationship?** | What positive developments regarding the patient-physician relationship do you see through pre-informed patients?  What negative developments regarding the patient-physician relationship due to pre-informed patients? | Here the physician should describe how the patient-physician  patient relationship has changed from their point of view  due to by digital media pre-informed patients.  Identification of the advantages and disadvantages from the physician's point of view. |
| IV | *Digital Media* | | |
|  | **To what extent do you recommend digital median to your patient during a consultation for their condition.** |  | Identification if physicians recommend digital media (website, platforms, apps etc.) to their patients.  Identification of reputable and/or quality-assured  digital information channels and  -sources from a physician's point of view. |
| V | *Patient Journey* | | |
|  | **In your opinion, how do digital information services influence the patient's journey through all phases of his or her illness?** | Can you describe if pre-informed patients delay seeking specialist care due to misinformation or misinterpretation of their research?  How often do you experience that patients after their search for information with a self-diagnosis or a therapy suggestion come to you?  To what extent do they think access to health care is affected by digital media use? | Identification of the influence of digital media on the patient journey from the physician’s view. |
| VI | *Trust* | | |
|  | **Can you describe how the use of digital media influence the relationship between you and the patients?** | Can you describe to what extent pre-informed patients question your opinion?  Could you observe changed in the therapy adherence among pre-informed patients (improved/worsened)? | Identification of possible effects of the  digital pre-informed patients on the patient-physician relationship and the therapy adherence of. |
| VII | *Chances and Risks* | | |
|  | **Looking into the near**  **future, what opportunities**  **and risks do you see**  **(1) on the one hand for digital**  **tools in the**  **healthcare system and**  **(2) on the other hand, for digital**  **information channels?** |  | Looking to the future:  Identification of opportunities and risks of digital  information channels and digital tools in the healthcare system from the perspective of physicians. |

We have now reached the end of the interview. Thank you for sharing your experience with us and thank you very much for your participation.
