# Supplementary material for: Impact of Digital Media on the Patient Journey and Patient-Physician Relationship Among Dermatologists and Adult Patients With Skin Diseases: Qualitative Interview Study
Source: J Med Internet Res. 2023 Sep 22;25:e44129. doi: 10.2196/44129 (PMC10559188; doi:10.2196/44129)
Supplement: Multimedia Appendix 3 [file jmir_v25i1e44129_app3.docx]

Supplement – Tables

**Table S1.** Items of interest derived from the interview guide for patients with skin diseases addressing digital media use in relation to the stage of the dermatological patient journey including opportunities and risks

| Stage of Patient Journey | Items of interest |
| --- | --- |
| Before consultation | Detection of skin disease signs or symptoms  Identification of information channels  Subjective judgment of health information available  Pattern of timely use  Motivation to search  Emotional situation following online search  Decision to consult a dermatologist |
| During consultation | Patient-Physician-Relationship  Factors influencing selection of dermatologist and appointment  Health information discussed with the dermatologist  Dermatologists position towards digital sources of information  Professional recommendation of suitable media  Effect of patient’s pre-information on communication |
| After consultation | Subjective perception by the patients  Relationship of trust towards dermatologist vs. digital media  Patients’ intent for post-consultation Internet search,  e.g., second opinion, alternative therapy options |
| Opportunities and risks | Quality of information and expectations |

**Table S2.** Items of interest derived from the interview guide for dermatologists addressing patient digital media regarding patient-physician interactions

| Dermatologists’ perspective | Items of interest |
| --- | --- |
| Physician role | Impact and possibly change |
| Physician judgement | Interaction with pre-informed patients  Patients’ motivation for Internet search  Factors favoring pre-consultation search of information  Patients’ emotions triggered by Internet pre-information  Patients e-health literacy  Information sources regarding quality  Recommendations for patients |
| Patient-physician relationship | Effect on relationship of trust towards dermatologists’  Effect on adherence to therapy |
| Effects on patient journey | Anamnestic exploration on - use of digital media  - self-diagnosis and self-therapy - belated consultation as a consequence of misinformation  - use of digital services, e.g., online appointments,  video consultation, electronic patient record |
| Dermatologist outlook | Opportunities and risks |
